# Microtubule array observed in the posterior‐vegetal cortex during cytoplasmic and cortical reorganization of the ascidian egg

**DOI:** 10.1111/dgd.12405

**Published:** 2017-10-02

**Authors:** Hirokazu Ishii, Toshiyuki Goto, Takahito Nishikata

**Affiliations:** ^1^ Frontiers of Innovative Research in Science and Technology (FIRST) Konan University Kobe Hyogo 650‐0047 Japan; ^2^ Present address: Research Institute for Electronic Science Hokkaido University Sapporo Hokkaido 001‐0020 Japan

**Keywords:** ascidian, axis formation, cell polarity, cortical microtubule array, maternal factors

## Abstract

Body axis formation during embryogenesis results from asymmetric localization of maternal factors in the egg. Shortly before the first cleavage in ascidian eggs, cell polarity along the anteroposterior (A–P) axis is established and the cytoplasmic domain (myoplasm) relocates from the vegetal to the posterior region in a microtubule‐dependent manner. Through immunostaining, tubulin accumulation during this reorganization is observable on the myoplasm cortex. However, more detailed morphological features of microtubules remain relatively unknown. In this study, we invented a new reagent that improves the immunostaining of cortical microtubules and successfully visualized a parallel array of thick microtubules. During reorganization, they covered nearly the entire myoplasm cortical region, beneath the posterior‐vegetal cortex. We designated this microtubule array as CAMP (cortical array of microtubules in posterior vegetal region). During the late phase of reorganization, CAMP shrank and the myoplasm formed a crescent‐like cytoplasmic domain. When the CAMP formation was inhibited by sodium azide, myoplasmic reorganization and A–P axis formation were both abolished, suggesting that CAMP is important for these two processes.

## Graphical abstract

We invented a new reagent that improves the immunostaining and successfully visualized a parallel array of thick microtubules in the posterior‐vegetal cortex of fertilized ascidian eggs. Our results suggest that the microtubule array is important for cytoplasmic and cortical reorganization and anteroposterior axis formation.

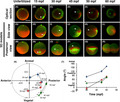

## Introduction

Asymmetric localization of maternal factors in the egg results in clear cell polarity, essential for body axis formation during embryogenesis. In some animal eggs, drastic cytoplasmic reorganization accompanies maternal‐factor localization, establishing cell polarity during the first cell cycle. In frog and zebrafish eggs, for example, highly aligned microtubule arrays appear transiently at the vegetal cortex during maternal‐factor reorganization; these microtubules are necessary for fixing egg polarity along the dorsoventral (D–V) axis (Elinson & Rowning 1988; Jesuthasan & Strähle 1997). However, the exact mechanisms underlying egg polarity establishment and cytoplasmic rearrangement remain unclear.

Ascidian eggs are a typical mosaic type with clearly distinguishable cytoplasmic regions, including the myoplasm, a mitochondria‐rich cytoplasmic domain (Conklin 1905). The endoplasmic reticulum and maternal mRNA (i.e. Type I *postplasmic/PEM* RNA) (Paix *et al*. 2009; Makabe & Nishida 2012) are localized in the cortical region of the myoplasm (Sardet *et al*. 2003; Nishida 2005; Prodon *et al*. 2007). The myoplasm exhibits obvious cell polarity along the animal‐vegetal (A–V) axis, being localized to the periphery (except for a small patch on the animal pole) in unfertilized eggs and shifting immediately to the vegetal hemisphere post‐fertilization. This first phase of cytoplasmic and cortical reorganization is microfilament‐dependent (Sawada & Schatten 1989; Chiba *et al*. 1999; Roegiers *et al*. 1999). The microtubule‐dependent (Sawada & Schatten 1989; Chiba *et al*. 1999; Roegiers *et al*. 1999) second phase begins approximately 30 min post‐fertilization, when the sperm aster moves from the vegetal cortex, reaching the equatorial region before turning toward the egg center. Sperm‐aster movement is accompanied by myoplasm formation into a crescent‐like cytoplasmic region, establishing the posterior side and therefore the anteroposterior (A–P) axis (Sardet *et al*. 1989; Roegiers *et al*. 1999). The strong correlation between cytoplasm and sperm‐aster movements suggested that the latter is responsible for the second segregation (Jeffery & Meier 1983; Jeffery & Swalla 1990), although previous immunostaining experiments have detected only obscure signs of tubulins in the posterior‐vegetal cortex (Chiba *et al*. 1999; Roegiers *et al*. 1999; Ishii *et al*. 2014). These relatively weak imaging results prevent us from clearly following myoplasmic movement and thus hamper attempts to clarify regulatory mechanisms of cytoplasmic rearrangement and body‐axis determination.

Here, we aimed to improve microtubule visualization through the development of a new immunostaining reagent. Our objective was to provide clear microtubule images during the second phase of reorganization on the posterior‐vegetal cortex. Additionally, we tested the involvement of microtubules in myoplasmic reorganization and A–P axis formation.

## Materials and methods

### Embryos

Ascidian (*Ciona intestinalis*) adults were provided by the National Bio‐Resource Project (NBRP), Japan. Egg and sperm handling, dechorionation, and fertilization followed previous publications (Ishii *et al*. 2012, 2014). Embryos were reared in filtered seawater at 18°C. At this temperature, the first and second reorganization phases occur immediately post‐fertilization and about 30 min post‐fertilization (mpf), respectively, while the first cleavage occurs around 60 mpf. The second phase was inhibited by treatment with 5 mmol/L sodium azide (NaN_3_) from 10 mpf. NaN_3_ prevents reorganization without depolymerizing microtubules (Ishii *et al*. 2014).

### Immunostaining

Whole *Ciona* embryos were fixed with 100% methanol, followed by 100% ethanol treatment. Fixed specimens were washed with phosphate‐buffered saline containing 0.05% Tween 20 (PBST), then treated with modified Sca*l*eA2 (G1T0; 4 mol/L urea [MP Biomedicals, Solon, OH, USA] and 1% glycerol in distilled water) for 90 min at 4°C, followed by a PBST wash. This treatment did not optically clear the specimens, but led to a sufficient improvement of microtubule immunostaining in the ascidian embryo cortex.

The G1T0‐treated specimens were stained with the following antibodies: anti‐*α*‐tubulin mouse monoclonal antibody (1:100 dilution, anti‐microtubule; CLT9002; Cedarlane Laboratories, Hornby, ON, Canada), anti‐MnSOD rabbit antisera (1:40 dilution, anti‐myoplasm; Ishii *et al*. 2012; SPC‐117C/D; StressMarq Biosciences, Victoria, Canada), Alexa Fluor 488‐conjugated goat anti‐mouse IgG antibody (1:1000 dilution, A11001; Molecular Probes, Eugene, OR, USA), and Alexa Fluor 532‐conjugated goat anti‐rabbit IgG antibody (1:1000 dilution, A11009; Molecular Probes). Stained specimens were cleared with methyl salicylate (Nacalai Tesque, Kyoto, Japan) and observed under a LSM700 confocal microscope (Carl Zeiss, Jena, Germany). Three‐dimensional images were rendered from confocal images, and mid‐plane images were generated in ZEN (Carl Zeiss).

### Image analysis

All analyses were performed in ImageJ (http://imagej.nih.gov/ij/). The egg contour was delineated and its central coordinates calculated from a side‐view of the 3D model. The animal pole was defined based on polar body or myoplasm configuration (depending on which was clearer); the image was then rotated to place the animal pole on top for generating the mid‐plane view. Sperm‐aster position was described based on centrosome coordinates. Myoplasm thickness was measured using the radial line profile per 10°, and its center of mass was calculated. The position of thick microtubule bundles on the posterior vegetal cortex was set as the midpoint of the circular arc formed by the bundles in the side view, equidistant from the upper‐ and lower‐most points of the structures. These positions were determined from three images per time point (30, 45, and 50 mpf), and standard deviations (SDs) were calculated. Microtubule movement was represented in two ways. First, each coordinate was directly plotted on a graph representing the egg's mid‐plane; second, the time course of angles between the animal‐vegetal axis and the line from the egg's center to each coordinate was plotted.

## Results

### Immunostaining optimization for cortical microtubules

We optimized immunostaining via modifying Sca*l*eA2 reagents to clarify cortical microtubule structures in *C*. *intestinalis* eggs. The Sca*l*eA2 reagent (4 mol/L urea, 10% glycerol, and 0.1% Triton X‐100 in water) is an optical clearing reagent for whole mount specimens (Hama *et al*. 2011). While, we found that the immunostaining of cortical microtubules in the fixed *Ciona* embryo was improved, when the modified Sca*l*eA2 was used as a pretreatment reagent.

**Figure 1:**
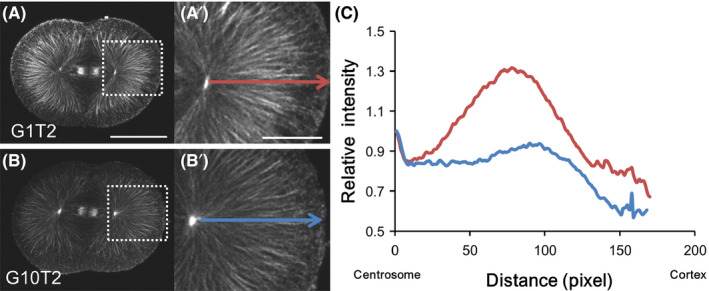
Evaluation of glycerol concentration in modified Sca*l*eA2 reagents. *Ciona* embryos fixed at 60 min post‐fertilization (mpf) were treated with modified Sca*l*eA2 reagents (Table 1) and immunostained for microtubule visualization. (A, B) Horizontal optical sections at the equatorial region of each specimen were compared. Photographs were taken at the same exposure. Scale bar, 50 µm. (A′, B′) Enlargement of the indicated region (white rectangle) in A and B, respectively. Scale bar, 20 µm. (C) Tubulin staining intensity was evaluated. Average fluorescent intensities of 100‐pixel widths along the indicated arrows were calculated. These graphs represent the fluorescent intensity relative to the centrosome region (basal point of arrows). G10T2 showed faint microtubule staining, whereas G1T2 staining was stronger. (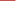 G1T2; 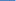, G10T2).

**Figure 2:**
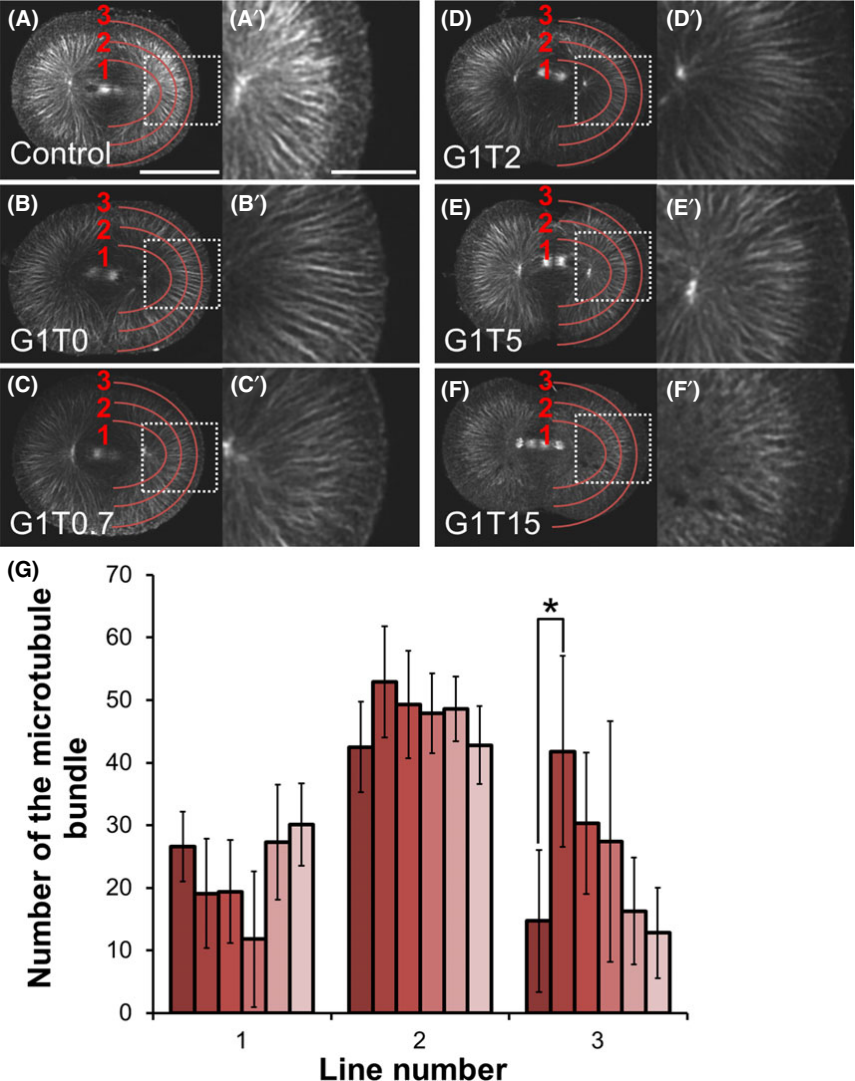
Evaluation of Triton X‐100 concentration in modified Sca*l*eA2 reagents. *Ciona* embryos fixed at 60 mpf were treated with modified Sca*l*eA2 reagents (Table 1) and immunostained for microtubule visualization. (A–F) Horizontal optical sections at the equatorial region of each specimen were compared. Control embryos were not treated with any Scale reagents. All photographs were taken with the same exposure. Scale bar, 50 µm. (A′–F′) Enlargement of the indicated region (white rectangle) in A–F. Scale bar, 20 µm. (G) Comparison of microtubule bundles detected along the indicated red lines. The G1T0 reagent caused a significant increase in the number of microtubule bundles in the outermost region (*P < 0.001). Error bars represent SDs (*n* = 6). (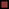, Control; 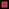, G1T0; 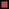, G1T0.7; 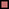, G1T2; 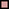, G1T5; 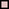 G1T15).

The optimal Sca*l*eA2 recipe was determined through testing a series of glycerol and Triton X‐100 concentrations (Table 1) using embryos fixed just before the first cleavage. Optimal glycerol concentrations were measured based on microtubule fluorescent intensity. While 10% glycerol diminished overall microtubule staining, 1% glycerol was sufficient for visualizing astral microtubules in the cortical region (Fig. 1). Next, optimal Triton X‐100 concentrations were measured based on the number of microtubule bundles observed on three lines at different depths (Fig. 2; lines 1 to 3). In the control embryo, which was not treated with Sca*l*e reagents (non‐treated), microtubule staining showed three faintly different layers according to the cytoplasmic depth (Fig. 2A). These lines are drawn in the middle of these three layers. While variation in Triton X‐100 concentration did not affect microtubule number on lines 1 and 2, higher Triton X‐100 concentrations diminished cortical tubulin staining on line 3. Thus, the modified reagent (G1T0) containing 4 mol/L of urea and 1% glycerol was the most efficient at visualizing microtubule structures in the cortical region.

**Table 1:** Composition of modified ScaleA2 reagents

Modified ScaleA2	Urea^a^	Triton X‐100^a^ (%)	Glycerol^a^(%)
Original	4.0 mol/L	0.1	10.0
G10T2	4.0 mol/L	2.0	10.0
G1T0	4.0 mol/L	0.0	1.0
G1T0.7	4.0 mol/L	0.7	1.0
G1T2	4.0 mol/L	2.0	1.0
G1T5	4.0 mol/L	5.0	1.0
G1T15	4.0 mol/L	15.0	1.0

a Urea, Triton X‐100 (w/v), and glycerol (w/v) were dissolved in distilled water.

### Cortical microtubule array in posterior‐vegetal region

**Figure 3:**
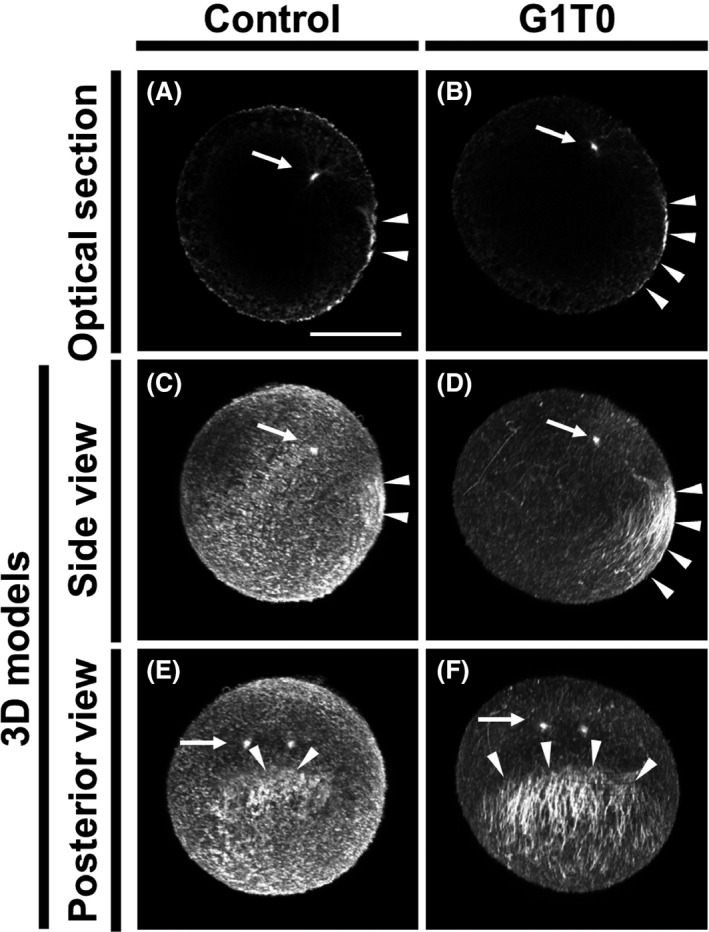
Cortical array of microtubules in the posterior‐vegetal region. *Ciona* one‐cell embryos at the second phase of reorganization (45 mpf) were stained to visualize microtubules. (A, C, E) Control specimen (non‐treated). (B, D, F) G1T0‐treated specimen. In all photographs, the animal pole is on the top. (A, B) Mid‐plane optical sections. Posterior pole is on the right. (C–F) 3D models rendered from confocal images. (C, D) Side views. Posterior pole is on the right. (E, F) Posterior views. Sperm asters and cortical microtubule arrays are indicated with arrows and arrowheads, respectively. Scale bar, 50 µm.

At 45 mpf in non‐treated specimens, a faint patch of tubulin staining was observed in the posterior‐vegetal cortex, confirming previous reports (Fig. 3A, C, E; Chiba *et al*. 1999; Roegiers *et al*. 1999; Ishii *et al*. 2014). In contrast, when the specimens were treated with G1T0, we observed thick microtubule bundles positioned in parallel arrays to the mid‐plane arc (Fig. 3B, D, F).

**Figure 4:**
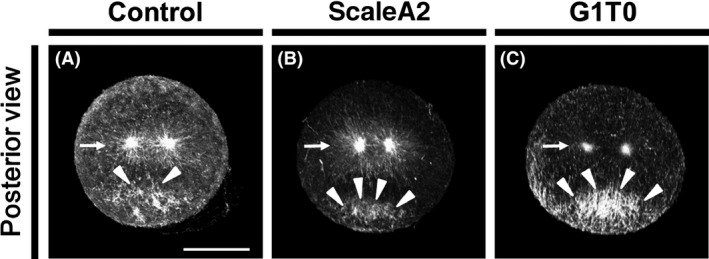
Comparison of effects of Scale reagents. *Ciona* one‐cell embryos at 45 mpf were stained to visualize microtubules. (A) Non‐treated control specimen. (B) Original ScaleA2 treated specimen. (C) G1T0‐treated specimen. (A–C) Posterior views of 3D models with the animal pole on the top. Sperm asters and cortical microtubule arrays are indicated with arrows and arrowheads, respectively. All photographs were taken and processed under the same condition. Scale bar, 50 µm.

The effects of original and modified Sca*l*e reagents were compared using embryos at 45 mpf (Fig. 4). In non‐treated specimens, the microtubule array in the posterior‐vegetal cortex was hardly observed, while the sperm aster was evidently observed (Fig. 4A). In the specimens treated with original Sca*l*eA2, overall microtubule staining was similar to the non‐treated specimens, while the background is lower than that of non‐treated specimens (Fig. 4B). In contrast, G1T0 treated embryo showed clear microtubule array on the posterior‐vegetal cortex, however, the sperm aster was appeared to be smaller than that in the non‐treated control and the original Sca*l*eA2 treated specimens (Fig. 4C).

### Changes in cortical microtubule structures during cytoplasmic and cortical reorganization

**Figure 5:**
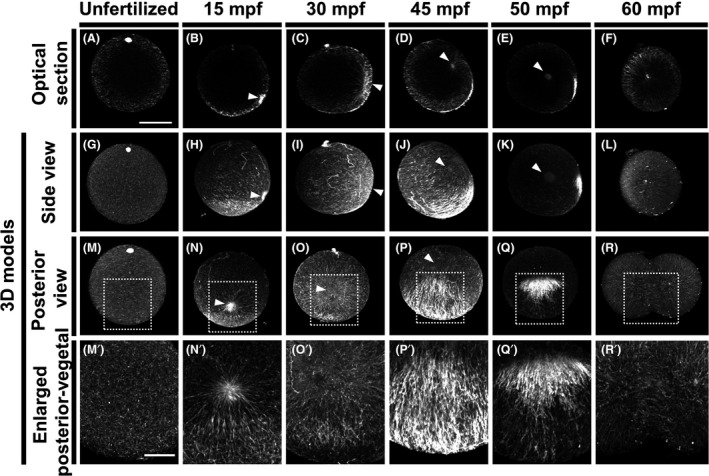
Changes in microtubule structure during cytoplasmic and cortical reorganization. *Ciona* eggs during the first cell cycle were stained for microtubules using G1T0. In all photographs, the animal pole is on top. The posterior pole is on the right in mid‐plane optical sections and 3D model side views. In the unfertilized egg (radial symmetry) and the 15‐mpf embryo (posterior side unclear), corresponding views are simply perpendicular to the tentatively assigned posterior view. (A, G, M, M′) Unfertilized egg. (B, H, N, N′) 15 mpf. (C, I, O, O′) 30 mpf. (D, J, P, P′) 45 mpf. (E, K, Q, Q′) 50 mpf. (F, L, R, R′) 60 mpf. (A–F) Mid‐plane optical sections with posterior pole on right. (G–R) 3D models. (G–L) Side views with posterior pole on right. (M–R) Posterior views. (M′–R′) Enlargement of the indicated region (white rectangle) in M–R. Arrowheads indicate sperm asters. Scale bars, 50 µm in A and 20 µm in M’.

In unfertilized G1T0‐treated eggs, short and faint microtubule fragments were observed beneath the cortex (Fig. 5A, G, M, M′). At 15 mpf, the dome‐like sperm aster appeared on the vegetal cortex (Figs. 5B, H, N); microtubule fragments accumulated there as well, forming a coarse meshwork to which some astral microtubules connected (Fig. 5N′). At 30 mpf, the now larger and spherical sperm aster had already migrated to the posterior equatorial region, after which the microtubule meshwork also shifted posteriorly (Fig. 5C, I, O, O′). At 45 mpf, obviously visible thick microtubules formed a parallel array beneath the posterior‐vegetal cortex (Fig. 5D, J, P, P′) that covered nearly the entire region. By this point, the sperm aster had shifted toward the egg center. Although previous reports have suggested that astral microtubules and cortical tubulin accumulation form a continuous structure (Chiba *et al*. 1999; Roegiers *et al*. 1999), we did not observe a strong connection between them at 45 mpf. At 50 mpf, the cortical microtubule array became shorter and primarily localized to the posterior (Figs. 5E, K, Q, Q′). Furthermore, cytoplasmic and cortical reorganization was complete, and cell polarity along the A–P axis was established. At 60 mpf, the cortical microtubule array had dissipated, and the mitotic apparatus had begun initial cell division in the center (Fig. 5F, L, R, R′).

The cortical microtubule array observed at 45 and 50 mpf was a novel structure that we designated as CAMP (cortical array of microtubules in posterior‐vegetal region). Its formation appears to occur after 30 mpf and is present by 45 mpf at the latest. The accumulation of fragmented microtubules (15 mpf) and subsequent meshwork in the vegetal cortex (30 mpf) might be precursors to CAMP.

### Correlation between myoplasm reorganization and microtubule structures

To understand whether the sperm aster or the CAMP microtubule system is involved in myoplasm movement, we double‐stained microtubules and myoplasm in G1T0‐treated specimens.

**Figure 6:**
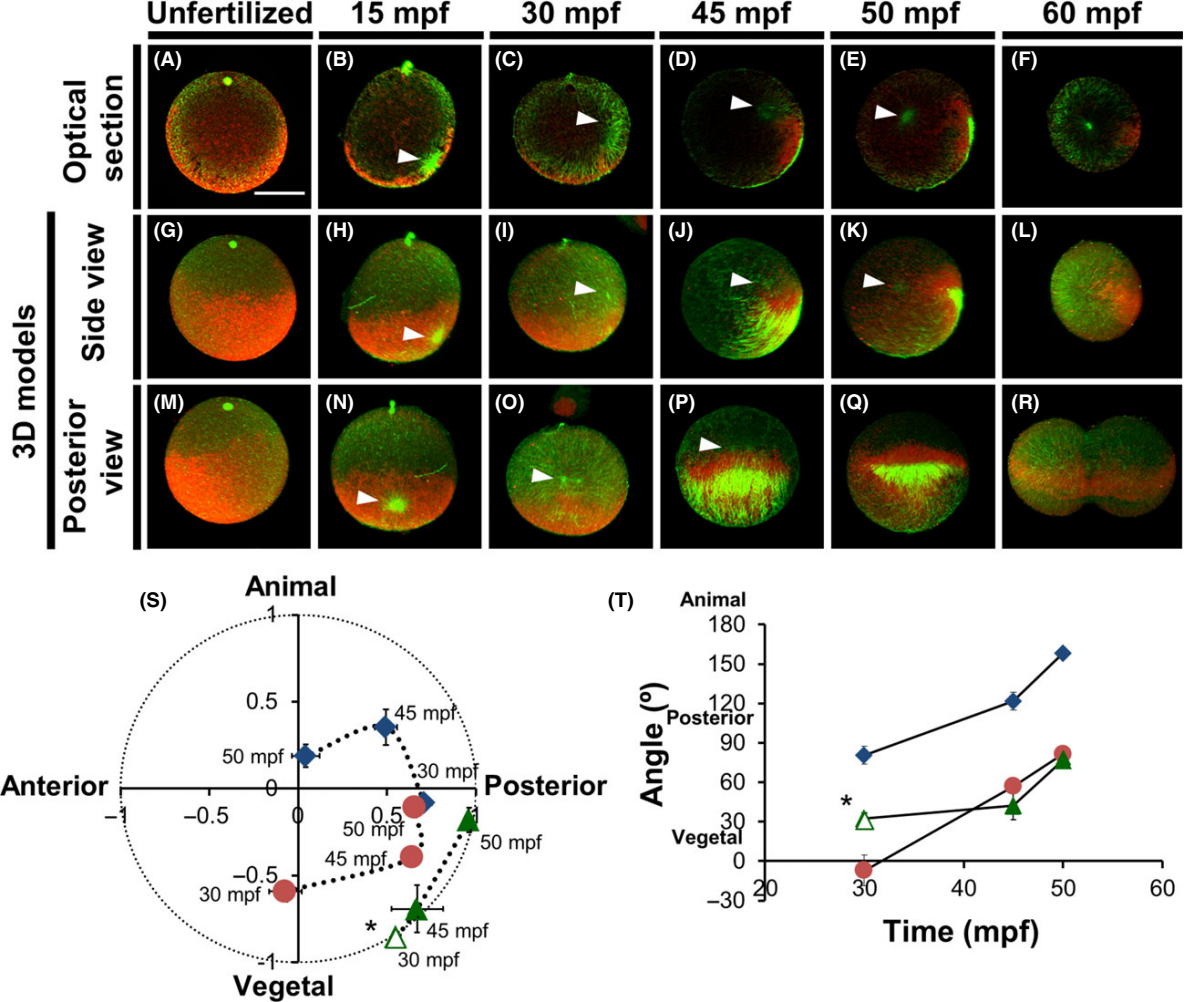
Correlations between microtubule structures and myoplasm reorganization. *Ciona* eggs during the first cell cycle were double‐stained for microtubules (green) and myoplasm (red) using G1T0. In all photographs, the animal pole faces upward. In mid‐plane optical sections and 3D‐model side views, the posterior pole is on the right. In the unfertilized egg (radial symmetry) and the 15‐mpf embryo (posterior side unclear), corresponding views are simply perpendicular to the tentatively assigned posterior view. (A, G, M) Unfertilized egg. (B, H, N) 15 mpf. (C, I, O) 30 mpf. (D, J, P) 45 mpf. (E, K, Q) 50 mpf. (F, L, R) 60 mpf. (A–F) Mid‐plane optical sections. (G–R) 3D models. (G–L) Side views. (M–R) Posterior views. Arrowheads indicate sperm asters. Scale bar, 50 µm. (S, T) Analysis of sperm aster, myoplasm, and microtubule movements from 30 to 50 mpf. Plots include the coordinates of the sperm‐aster centrosome (♦), myoplasm center of mass (●), and the middle of the cortical microtubule meshwork (*▵; 30 mpf) and CAMP (▲; 45 mpf, 50 mpf). Error bars represent SDs (*n* = 3). (S) Vertical and horizontal axes represent animal‐vegetal and anteroposterior axes, respectively. The origin of the coordinate axes is the egg center, and the egg radius is represented as 1.0 (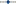, Sperm aster; 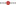 Myoplasm; 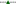, CAMP). (T) Movement time courses are represented as the angle (+90° to −90°) from the animal‐vegetal axis (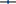, Sperm aster; 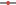
Myoplasm; 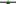, CAMP).

The myoplasm of the unfertilized egg was localized only in the outermost layer of the cortical cytoplasm (except at the animal pole), whereas the microtubule meshwork spread deeper in the peripheral cytoplasm (Fig. 6A, G, M). At 15 mpf, the microtubule meshwork was restricted to the vegetal pole, while the myoplasm was more widely distributed on the vegetal cortex (Fig. 6B, H, N), with the sperm aster nearby. At 30 mpf, the myoplasm remained at the vegetal hemisphere, but the sperm aster had separated from the myoplasm and moved to the equatorial region (Fig. 6C, I, O). At 45 mpf, CAMP formed from densely aligned cortical microtubules, covering nearly the entire cortex of the myoplasm (except the equatorial region), which had moved to the posterior‐vegetal region (Fig. 6D, J, P) while the sperm aster had begun moving toward the egg center. At 50 mpf, the sperm aster was localized to the egg center and CAMP had shrunk into the posterior pole within the myoplasm, which moved posteriorly to form a crescent‐like cytoplasmic domain (Fig. 6E, K, Q). Mitosis began at 60 mpf; myoplasm rearrangement in the posterior region was complete, and CAMP was no longer detectable (Fig. 6F, L, R). The appearance and shrinkage of CAMP around 45 mpf at the posterior‐vegetal cortex coincided with the second phase of reorganization.

When we analyzed the relationship between myoplasm and microtubule structures through mapping their relative positions (see Materials and Methods), we found that the sperm aster was positioned far ahead of the myoplasm during 30–50 mpf (Fig. 6S). Furthermore, myoplasm movement was accompanied by CAMP shrinkage from 45 mpf to 50 mpf. This correlation was confirmed with similarities in the angle trajectories between the A–V axis and the lines from the egg center to the myoplasm center or the CAMP midpoint (Fig. 6T). Overall, the data suggest that CAMP shrinkage during 45–50 mpf contributes to myoplasm movement. However, the CAMP shrinkage solely might not drive the movement of the subcortical myoplasm to form a crescent like cytoplasmic domain. Although we did not observe direct contact between sperm astral microtubules and CAMP, we feel confident in proposing that the direction of sperm‐aster movement and subsequent CAMP formation defines the A–P axis.

### Myoplasm reorganization failed in CAMP‐disturbed eggs

**Figure 7:**
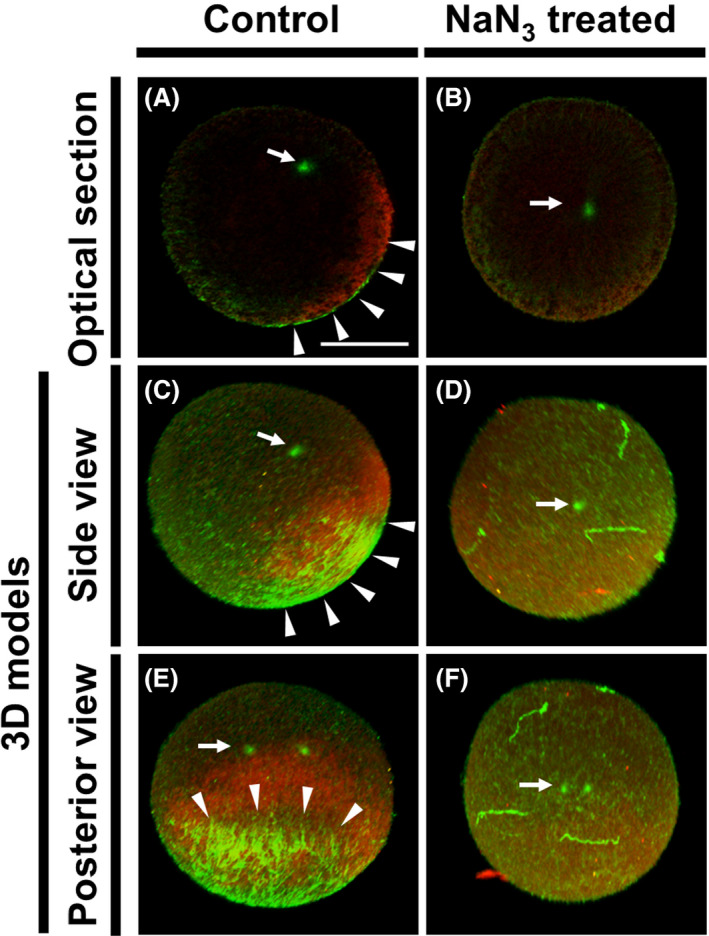
The effects of NaN_3_ on CAMP and myoplasm reorganization. *Ciona* embryos were treated with NaN_3_ (5 mM) during the second phase of reorganization and fixed at 45 mpf. Specimens were stained for microtubules (green) and myoplasm (red) using G1T0. In all photographs, the animal pole is oriented upward. (A, C, E) Control embryo without NaN_3_‐treatment. (B, D, F) NaN_3_‐treated embryo. (A, B) Mid‐plane optical sections. (C–F) 3D models. (C, D) Side views with posterior pole on the right. (E, F) Posterior views. Arrows and arrowheads indicate sperm‐aster centrosome and CAMP, respectively. Scale bar, 50 µm. Note that in the NaN_3_‐treated embryo, CAMP is not visible and myoplasm is not asymmetric localized, despite the sperm aster being in the same location as in the control embryo.

Previously, we reported that NaN_3_ treatment during the second phase of reorganization disturbed cortical tubulin accumulation but not astral microtubule structure (Ishii *et al*. 2014), suggesting a specific inhibition of CAMP formation. So we visualized the effects of NaN_3_ on the CAMP formation by immunostaining using G1T0 (Fig. 7). The results of NaN_3_ treatment on embryos indicated that CAMP was abolished, but the sperm aster remained in the egg center (Fig. 7B). Additionally, the myoplasm did not move to the posterior region and the A–P axis was not observed (Fig. 7; Ishii *et al*. 2014). On the other hand, the sperm aster was appeared to be smaller than that in our previous study (Ishii *et al*. 2014), because the G1T0 treated embryo has small sperm aster compared to the untreated embryo as shown in Figure 4.

## Discussion

In this study, we optimized microtubule immunostaining using a modified Sca*l*eA2 reagent and described the presence of CAMP during the second phase of reorganization in the ascidian egg. While Sca*l*eA2 was typically used as an optical clearing reagent (Hama *et al*. 2011), our modified sca*l*e reagent was used for the pretreatment of immunostaining and successfully improved the immunostaining of thick microtubule structures on the cell cortex. The G1T0 reagent was very simple to formulate and effective in *Ciona*; thus, it might be useful for clarifying cortical microtubule structures in eggs and embryos of other species.

Previous researchers had considered that sperm‐aster microtubules were responsible for myoplasm movement during the second phase of ascidian‐egg reorganization, because it was the only microtubule structure thought to be present during that period (Sawada & Schatten 1988; Sardet *et al*. 1989, 2007). However, in this study, we successfully visualized CAMP, demonstrating that its movements were coordinated with those of the myoplasm. In addition, we showed that CAMP is responsible for myoplasm movement, as abolishing the former arrested the latter. Moreover, similar to the sperm aster movement, CAMP is likely also essential for determining the posterior pole because the second phase establishes the A–P axis.

During the second reorganization phase, two types of microtubule‐dependent translocations occur in the ascidian *Phallusia mammillata* (Sardet *et al*. 1989; Roegiers *et al*. 1999). The first is a slow translocation (25–40 mpf) associated with posterior cortex oscillation accompanying female pronucleus migration. The second is a fast translocation (40–45 mpf) characterized by movement of the duplicated male centrosome, asters, and myoplasm toward the egg center. Given the timing, our results suggest that myoplasm‐associated CAMP shrinkage to the posterior pole (at 45 mpf and 50 mpf) may be responsible for fast translocation.

Due to the close association between CAMP and myoplasm movement, the former probably also influences microtubule‐dependent relocation of type I *postplasmic/PEM* RNA to the posterior cortex (Sasakura *et al*. 2000; Tanaka *et al*. 2004). These RNAs play pivotal roles in body axis formation and tissue differentiation during embryogenesis (Sardet *et al*. 2003; Prodon *et al*. 2007; Makabe & Nishida 2012). Additionally, CAMP may also affect the posterior transport of cortical endoplasmic reticulum (cER)‐mRNA domains. These structures form from the anchoring of some type I *postplasmic/PEM* RNA (e.g. *macho‐1*) to the cER (Sardet *et al*. 2003). Thus, studies of the interaction between the CAMP and type I *postplasmic/PEM* RNA or the cER should increase current understanding of mRNA transport mechanisms during the second phase of reorganization.

Although novel to ascidian eggs, similar microtubule structures to CAMP have been observed in other animals. For example, parallel arrays of microtubule bundles appear in the vegetal cortex of fertilized zebrafish eggs (Jesuthasan & Strähle 1997; Tran *et al*. 2012). This cortical microtubule array is responsible for maternal mRNA (e.g. maternal dorsalizing factor, *wnt8a*) transport (Tran *et al*. 2012; Ge *et al*. 2014). Microtubule array formation is mediated by Ca^2+^ signaling, but independent of sperm entry and fertilization (Tran *et al*. 2012). In fertilized frog eggs, a parallel microtubule array forms beneath the vegetal cortex during cortical rotation (a form of cytoplasmic reorganization) (Elinson & Rowning 1988; Houliston & Elinson 1992; Elinson & Ninomiya 2003), without requiring the presence of a sperm aster (Elinson & Rowning 1988). Microtubule plus ends indicate the future dorsal side, and a dorsal determinant candidate (disheveled protein) was reported to be translocated dorsally along the array (Miller *et al*. 1999; Weaver & Kimelman 2004). Including the newly reported CAMP in ascidian eggs, these arrays are similar to each other in the following aspects: (i) localized to the vegetal cortex, (ii) comprise parallel bundles extending along the future body axis, and (iii) contribute to maternal factor transport. These similarities strongly support the notion that the cortical microtubule array is a conserved structure in early development, functioning to relocate maternal factors for future body axis determination. Therefore, cross‐chordate comparisons of the microtubule array formations will contribute to our knowledge of conserved mechanisms in cytoplasm reorganization and body axis formation during embryogenesis.

## Author contribution

TN conceived and designed the study, and assisted in manuscript preparation. HI and TG performed the experiments and wrote the manuscript draft. All authors contributed to data analysis and interpretation, and critically reviewed the manuscript. The final version of the manuscript was approved by all authors.

## References

[dgd12405-bib-0001] Chiba, S. , Miki, Y. , Ashida, K. , Wada, M. R. , Tanaka, K. J. , Shibata, Y. , Nakamori, R. & Nishikata, T. 1999. Interactions between cytoskeletal components during myoplasm rearrangement in ascidian eggs. Dev. Growth Differ. 41, 265–272. 10400388 10.1046/j.1440-169x.1999.413433.x

[dgd12405-bib-0002] Conklin, E. G. 1905. The organization and cell lineage of the ascidian egg. J. Acad. Nat. Sci. Phila. 13, 1–119.

[dgd12405-bib-0003] Elinson, R. P. & Ninomiya, H. 2003. Parallel microtubules and other conserved elements of dorsal axial specification in the direct developing frog, *Eleutherodactylus coqui* . Dev. Genes. Evol. 213, 28–34. 12590350 10.1007/s00427-002-0290-8

[dgd12405-bib-0004] Elinson, R. P. & Rowning, B. 1988. A transient array of parallel microtubules in frog eggs: potential tracks for a cytoplasmic rotation that specifies the dorso‐ventral axis. Dev. Biol. 128, 185–197. 3289985 10.1016/0012-1606(88)90281-3

[dgd12405-bib-0005] Ge, X. , Grotjahn, D. , Welch, E. , Lyman‐Gingerich, J. , Holguin, C. , Dimitrova, E. , Abrams, E. W. , Gupta, T. , Marlow, F. L. , Yabe, T. , Adler, A. , Mullins, M. C. & Pelegri, F. 2014. Hecate/Grip2a acts to reorganize the cytoskeleton in the symmetry‐breaking event of embryonic axis induction. PLoS Genet. 10, e1004422. 24967891 10.1371/journal.pgen.1004422 PMC4072529

[dgd12405-bib-0006] Hama, H. , Kurokawa, H. , Kawano, H. , Ando, R. , Shimogori, T. , Noda, H. , Fukami, K. , Sakaue‐Sawano, A. & Miyawaki, A. 2011. Sca*l*e: a chemical approach for fluorescence imaging and reconstruction of transparent mouse brain. Nat. Neurosci. 14, 1481–1488. 21878933 10.1038/nn.2928

[dgd12405-bib-0007] Houliston, E. & Elinson, R. P. 1992. Microtubules and cytoplasmic reorganization in the frog egg. Curr. Top. Dev. Biol. 26, 53–70. 1563279 10.1016/s0070-2153(08)60440-8

[dgd12405-bib-0008] Ishii, H. , Kunihiro, S. , Tanaka, M. , Hatano, K. & Nishikata, T. 2012. Cytosolic subunits of ATP synthase are localized to the cortical endoplasmic reticulum‐rich domain of the ascidian egg myoplasm. Dev. Growth Differ. 54, 753–766. 23067137 10.1111/dgd.12003

[dgd12405-bib-0009] Ishii, H. , Shirai, T. , Makino, C. & Nishikata, T. 2014. Mitochondrial inhibitor sodium azide inhibits the reorganization of mitochondria‐rich cytoplasm and the establishment of the anteroposterior axis in ascidian embryo. Dev. Growth Differ. 56, 175–188. 24417477 10.1111/dgd.12117

[dgd12405-bib-0010] Jeffery, W. R. & Meier, S. 1983. A yellow crescent cytoskeletal domain in ascidian eggs and its role in early development. Dev. Biol. 96, 125–143. 6186551 10.1016/0012-1606(83)90317-2

[dgd12405-bib-0011] Jeffery, W. R. & Swalla, B. J. 1990. The myoplasm of ascidian eggs: a localized cytoskeletal domain with multiple roles in embryonic development. Semin. Cell Biol. 1, 373–381. 2102391

[dgd12405-bib-0012] Jesuthasan, S. & Strähle, U. 1997. Dynamic microtubules and specification of the zebrafish embryonic axis. Curr. Biol. 7, 31–42. 9024620 10.1016/s0960-9822(06)00025-x

[dgd12405-bib-0013] Makabe, K. W. & Nishida, H. 2012. Cytoplasmic localization and reorganization in ascidian eggs: role of postplasmic/PEM RNAs in axis formation and fate determination. Rev. Dev. Biol. 2012(1), 501–518. 10.1002/wdev.54 23801532

[dgd12405-bib-0014] Miller, J. R. , Rowning, B. A. , Larabell, C. A. , Yang‐Snyder, J. A. , Bates, R. L. & Moon, R. T. 1999. Establishment of the dorsal‐ventral axis in *Xenopus* embryos coincides with the dorsal enrichment of dishevelled that is dependent on cortical rotation. J. Cell Biol. 146, 427–437. 10427095 10.1083/jcb.146.2.427 PMC2156185

[dgd12405-bib-0015] Nishida, H. 2005. Specification of embryonic axis and mosaic development in ascidians. Dev. Dyn. 233, 1177–1193. 15973692 10.1002/dvdy.20469

[dgd12405-bib-0016] Paix, A. , Yamada, L. , Dru, P. , Lecordier, H. , Pruliere, G. , Chenevert, J. , Satoh, N. & Sardet, C. 2009. Cortical anchorages and cell type segregations of maternal postplasmic/PEM RNAs in ascidians. Dev. Biol. 336, 96–111. 19735652 10.1016/j.ydbio.2009.09.001

[dgd12405-bib-0017] Prodon, F. , Yamada, L. , Shirae‐Kurabayashi, M. , Nakamura, Y. & Sasakura, Y. 2007. Postplasmic/PEM RNAs: a class of localized maternal mRNAs with multiple roles in cell polarity and development in ascidian embryos. Dev. Dyn. 236, 1698–1715. 17366574 10.1002/dvdy.21109

[dgd12405-bib-0018] Roegiers, F. , Djediat, C. , Dumollard, R. , Rouvière, C. & Sardet, C. 1999. Phases of cytoplasmic and cortical reorganizations of the ascidian zygote between fertilization and first division. Development 126, 3101–3117. 10375502 10.1242/dev.126.14.3101

[dgd12405-bib-0019] Sardet, C. , Speksnijder, J. , Inoue, S. & Jaffe, L. 1989. Fertilization and ooplasmic movements in the ascidian egg. Development 105, 237–249. 2806123 10.1242/dev.105.2.237

[dgd12405-bib-0020] Sardet, C. , Nishida, H. , Prodon, F. & Sawada, K. 2003. Maternal mRNAs of PEM and macho 1, the ascidian muscle determinant, associate and move with a rough endoplasmic reticulum network in the egg cortex. Development 130, 5839–5849. 14573512 10.1242/dev.00805

[dgd12405-bib-0021] Sardet, C. , Paix, A. , Prodon, F. , Dru, P. & Chenevert, J. 2007. From oocyte to 16‐cell stage: cytoplasmic and cortical reorganizations that pattern the ascidian embryo. Dev. Dyn. 236, 1716–1731. 17420986 10.1002/dvdy.21136

[dgd12405-bib-0022] Sasakura, Y. , Ogasawara, M. & Makabe, K. W. 2000. Two pathways of maternal RNA localization at the posterior‐vegetal cytoplasm in early ascidian embryos. Dev. Biol. 220, 365–378. 10753523 10.1006/dbio.2000.9626

[dgd12405-bib-0023] Sawada, T. & Schatten, G. 1988. Microtubules in ascidian eggs during meiosis, fertilization, and mitosis. Cell Motil. Cytoskeleton 9, 219–230. 3365772 10.1002/cm.970090304

[dgd12405-bib-0024] Sawada, T. & Schatten, G. 1989. Effects of cytoskeletal inhibitors on ooplasmic segregation and microtubule organization during fertilization and early development in the ascidian *Molgula occidentalis* . Dev. Biol. 132, 331–342. 2466714 10.1016/0012-1606(89)90230-3

[dgd12405-bib-0025] Tanaka, K. J. , Matsumoto, K. , Tsujimoto, M. & Nishikata, T. 2004. CiYB1 is a major component of storage mRNPs in ascidian oocytes: implications in translational regulation of localized mRNAs. Dev. Biol. 272, 217–230. 15242802 10.1016/j.ydbio.2004.04.032

[dgd12405-bib-0026] Tran, L. D. , Hino, H. , Quach, H. , Lim, S. , Shindo, A. , Mimori‐Kiyosue, Y. , Mione, M. , Ueno, N. , Winkler, C. , Hibi, M. & Sampath, K. 2012. Dynamic microtubules at the vegetal cortex predict the embryonic axis in zebrafish. Development 139, 3644–3652. 22949618 10.1242/dev.082362

[dgd12405-bib-0027] Weaver, C. & Kimelman, D. 2004. Move it or lose it: axis specification in *Xenopus* . Development 131, 3491–3499. 15262887 10.1242/dev.01284

